# Non-isolated sources of electromagnetic radiation by multipole decomposition for photonic quantum technologies on a chip with nanoscale apertures†

**DOI:** 10.1039/d0na00580k

**Published:** 2020-10-08

**Authors:** Yuriy A. Artemyev, Vassili Savinov, Aviad Katiyi, Alexander S. Shalin, Alina Karabchevsky

**Affiliations:** School of Electrical and Computer Engineering, Ben-Gurion University Beer-Sheva Israel alinak@bgu.ac.il; Department of Nano-Photonics and Metamaterials, ITMO University St. Petersburg Russia; Optoelectronics Research Centre, Centre for Photonic Metamaterials, University of Southampton Southampton UK

## Abstract

The creation of single photon sources on a chip is a mid-term milestone on the road to chip-scale quantum computing. An in-depth understanding of the extended multipole decomposition of non-isolated sources of electromagnetic radiation is not only relevant for a microscopic description of fundamental phenomena, such as light propagation in a medium, but also for emerging applications such as single-photon sources. To design single photon emitters on a chip, we consider a ridge dielectric waveguide perturbed with a cylindrical inclusion. For this, we expanded classical multipole decomposition that allows simplifying and interpreting complex optical interactions in an intuitive manner to make it suitable for analyzing light-matter interactions with non-isolated objects that are parts of a larger network, *e.g.* individual components such as a single photon source of an optical chip. It is shown that our formalism can be used to design single photon sources on a chip.

## Abstract 2

The creation of single-photon sources on a chip with nanoscale apertures is a mid-term milestone on the road to chip-scale quantum computing.

## Graphical abstract

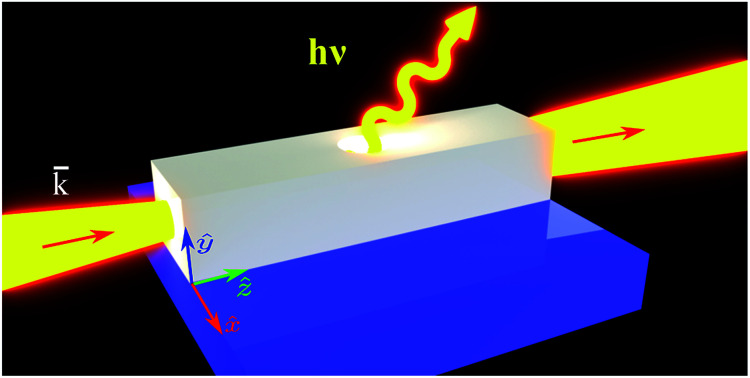

## 1 Introduction

Splitting complex systems, such as integrated circuits, into small, simple, but heavily interlinked parts, *i.e.* modularization, has over the last century enabled teams of engineers and scientists to pull their strengths together and tackle tasks of ever-increasing complexity.^1–5^ This type of collaborative work requires mathematical and numerical tools that would allow a researcher to develop and analyze non-isolated systems, in order to link them together. Nanophotonics is expected to be the key-enabling technology of this century, with application in biological sensing, DNA sequencing, high-speed information processing, quantum metrology, *etc.*^6–12^ Yet, as it stands now, modularization in nanophotonics is difficult to achieve. The main method for understanding interactions of light with nanoscale objects, multipole decomposition,^13^ is currently geared toward isolated systems, that is, isolated specks of matter suspended in a vacuum.^14,15^ Here, for the first time, we introduce systematic modifications to conventional multipole decomposition, which would enable analysis of isolated as well as non-isolated nanophotonic systems within a single framework. Our work paves the way for transition of nanophotonics from academia to industry.

Electromagnetic multipole expansion^13,16–20^ is a powerful tool for analyzing both the electric and magnetic fields created by spatially localized charges and currents.^17,18,21–25^ Electric multipoles are obtained by decomposing charge density, while magnetic (and toroidal) multipoles are obtained by decomposing currents.^26^ However, charge and current densities only remain independent in the static limit. In the electrodynamic case, charge and current densities become linked through charge conservation. Hence it becomes inconvenient to separate them for the purpose of multipole decomposition. In principle, this can be achieved by re-expressing electric multipoles in terms of current density (**J**).^26,27^ However, naive application of the widely cited formula can lead to errors in the case of non-isolated components of a device that has current flowing through it, *i.e.* the current density projection onto the surface normal (**n**) is non-zero (**n·J** ≠ 0). A typical example of a popular photonic system where such an effect arises is an optical waveguide.

When a disturbance of any shape is illuminated by the guided mode^1,28–31^ in a waveguide core (or by the evanescent field), the currents are no longer enclosed within the physical volume of this inclusion and, therefore, multipole decomposition needs to be modified. To date, this modification has not been addressed. Although multipole excitations have been studied extensively in isolated systems, such as all-dielectric nanoparticles,^32^ metasurfaces,^33^ molecules,^34^ and even discrete waveguides made of high-index dielectric nanoparticles,^35^ the excitation of multipoles in non-isolated systems, such as continuous waveguides, is still a puzzling question.

The contribution of the dipole moment (the first term after the monopole in the multipole series) to scattering was extensively studied with waveguides in relation to the Raman scattering effect.^36–38^ However, in these studies, the dipole moment was considered as a physical entity, rather than part of a multipole series. In addition, the contribution of higher-order multipole moments to scattering has not been explored with waveguide systems. Higher-order multipoles in dielectric particles are resonantly excited when the particle size increases with respect to the illumination wavelength. The far-field radiation patterns of single particles caused by high-order multipoles are different from those created by dipoles. Due to such resonant excitations – explained by the radiation patterns and, in other cases, the interplay between resonances of different orders – we can predict interesting properties of considered nanostructures, and these nanostructures can be utilized for a variety of applications, including nano-antennas,^39–41^ sensors,^29,42^ solar-cell technology,^43^ and multi-functional metamaterials.^44^

**Fig. 1:**
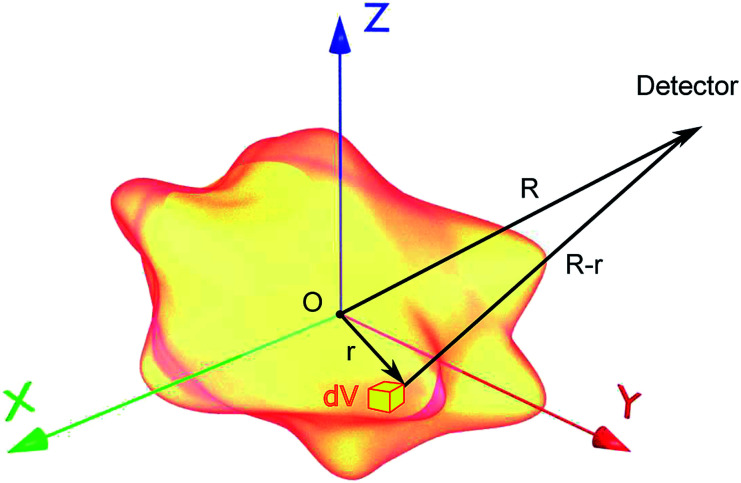
Particle of an arbitrary shape with volume *V* placed at the origin *O*. The particle represents the source of electromagnetic fields with charge density *ρ* and current density **J**. The observation point is shown as a detector at a distance *R* from the origin *O*; d*V* is the volume increment.

As was stressed above, the established classical multipole decomposition is not suited for electrodynamic systems where current density leaks through the boundaries (Fig. 1). Therefore, here we propose a new approach, which includes all the modifications to account for such surface effects.

The paper is organized in the following way: Section 2 describes the theory we developed. Proof-of-concept numerical results are presented in Subsection 2.2.2. In Section 3, we propose an example of realization of the developed approach and show the evolution of multipole moments when the inclusion diameter varies. We conclude the research in Section 4.

## 2 Multipole decomposition: analytical description

### 2.1 The amendments for open sources

An open source is a source whose parameters cannot be determined separately from the ambient state. The approach discussed below provides the possibility of significant reduction of the region that needs to be analysed. In this section, we consider an open system for which we propose amendments to classical multipole decomposition. For the general problem of rigorous multipole decomposition of an arbitrary portion of a given current density (or polarization), we present final relations while the detailed derivations are given in the ESI.† For the fields and the total intensity we obtain1
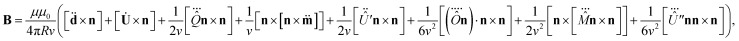
2
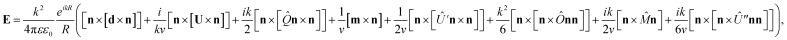
and3
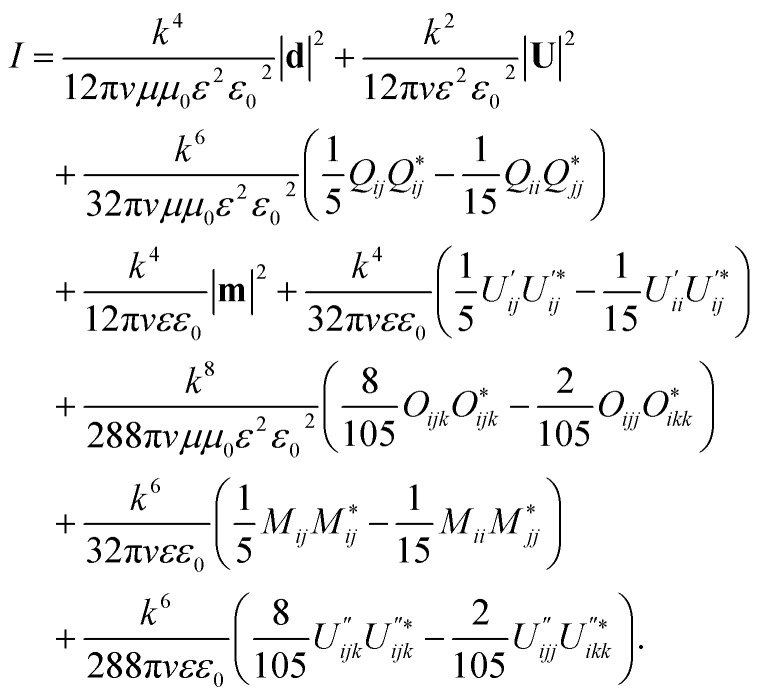


Different terms depend differently on the optical contrast of the medium *ε*. The notations in these equations refer to the following:

**n** is the unit vector in direction to an observation point, *d*_*i*_ is the electric dipole moment, *m*_*i*_ is the magnetic dipole moment, *Q*_*ij*_ is the electric quadrupole moment, *O*_*ijk*_ is the electric octupole moment, *M*_*ij*_ is the magnetic quadrupole moment, *U*_*i*_ is *i*^th^ component of an amendment vector obtained as the surface integral (see the ESI†), 
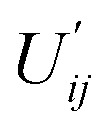
 is the component of an amendment of the second order, 
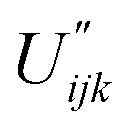
 is the component of an amendment of the third order.

Also, *v* is the light speed in the medium, *k* is the propagation coefficient in the medium and the overdot is the partial derivative over the retarded time.

We note that the model described above presents a modified implementation of the Babinet principle.^45^ Therefore, as was stated above, this allows for a reduction of the treated region. The modifications to the formulas are required because of the purely defined boundaries. This becomes obvious when considering an effective source, as in our example below. The above suggested approach provides an elegant solution also for numerical implementation. However, for more conventional numerical implementation we suggest the domain merging approach.

### 2.2 Domain merging approach

The main idea behind domain merging is to represent additional materials by introducing equivalent current distributions into the original ambient medium.

**Fig. 2:**
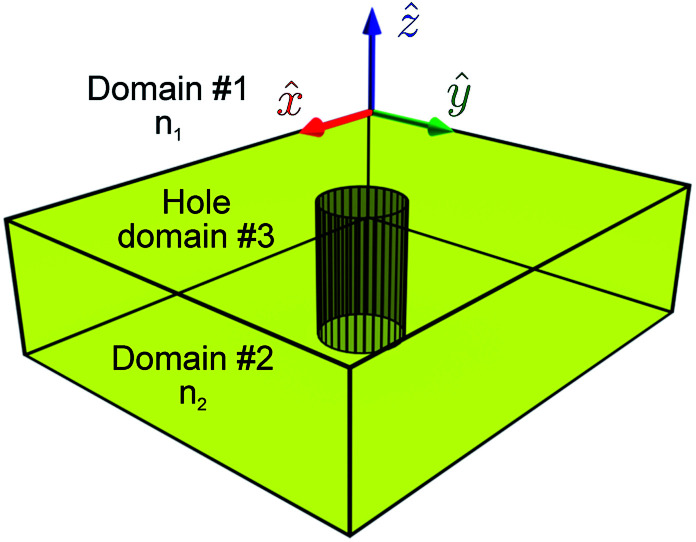
Illustration of the domain merging approach. Two dielectric half-spaces with refractive indices *n*_1,2_, respectively. The interface between the two domains lies in the *z* = 0 plane. There is a single inclusion in the *z* < 0 half-space labeled ‘hole’. This is a third domain with an arbitrary index of refraction.

The previous section focused on a very general problem of rigorous multipole decomposition of an arbitrary portion of a given current density (or polarization). In this section, we offer a simplified approach suited for simple geometries with few hard-to-handle inclusions. Here, we consider the reflection/transmission at the interface of two dielectric half-spaces where one of the two half-spaces has a small inclusion. The geometry is illustrated in Fig. 2.

The interface between the two half-spaces is in the *xy*-plane and is located at *z* = 0. The inclusion is a small hole in the *z* < 0 half-space. Without the hole, the problem would be solvable analytically using Fresnel equations. The presence of the hole will, in most cases, require numerical solvers.

Unfortunately, numerical solvers, whilst great at churning out solutions, often provide little insight into the problem at hand. We will now show how one can recover some of the intuitive simplicity offered by the multipole decomposition approach even in the case of such non-isolated problems as in Fig. 2. One starts by considering the solutions to Maxwell's equations in the three domains: the *z* > 0 half-space with refractive index *n*_1_ (domain #1), the *z* < 0 half-space with refractive index *n*_2_ (domain #2), and the hole with refractive index *n*(**r**) (domain #3). As the notation suggests, we do not limit the refractive index of the hole in any way, allowing it to depend on the position.

Assuming that the magnetic response of all the domains is trivial, the electric field in all three domains is given by the solution of the Helmholtz equation. Here we will use complex-harmonic fields with convention exp(−*iωt*), where *t* is the time and *ω* is the angular frequency. The three Helmholtz equations are:

4 (∇^2^ + *k*_0_^2^*n*_1_^2^)**E**_1_ = 0

5 (∇^2^ + *k*_0_^2^*n*_2_^2^)**E**_2_ = 0

6 [∇^2^ + *k*_0_^2^*n*(**r**)^2^]**E**_3_ = 0

where *k*_0_ is the free-space wavenumber. Our aim is now to merge domain #3 into domain #2, by converting eqn (6) into eqn (5). This is performed by moving all the unwanted parts of eqn (6) onto the right-hand side and treating it as a source:

7 [∇^2^ + *k*_0_^2^*n*(**r**)^2^]**E**_3_ = 0

8 (∇^2^ + *k*_0_^2^*n*_2_^2^)**E**_3_ = *k*_0_^2^[*n*_2_^2^ − *n*(**r**)^2^]**E**_3_

9 (∇^2^ + *k*_0_^2^*n*_2_^2^)**E**_3_ = −*iωμ*_0_**J**

Above we have defined the current density:

10 **J** ≡ −*iωε*_0_[*n*(**r**)^2^ − *n*_2_^2^]**E**

This definition can be extended over domain #2 as well as #3, since the current density identically disappears in the former. One can thus reduce the number of domains to just domain #1 and the extended second domain #2′:

11 (∇^2^ + *k*_0_^2^*n*_1_^2^)**E**_1_ = 0

12 (∇^2^ + *k*_0_^2^*n*_2_^2^)**E**_2′_ = −*iωμ*_0_**J**

One can go further still and show that solution in the domain #2′ is given by:13
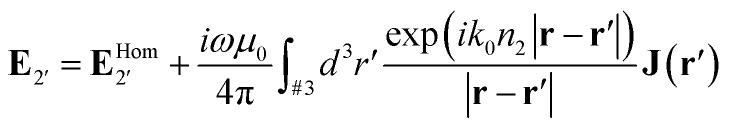
where 
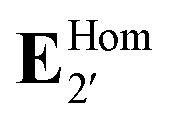
 is the homogeneous solution 
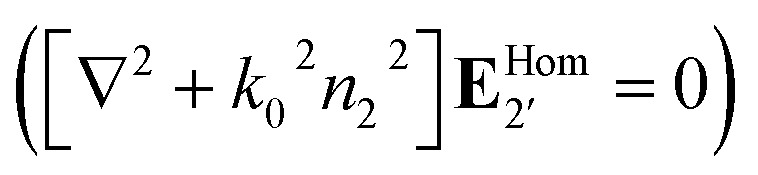
. At this point it is convenient to return to arbitrary time-dependence by applying the inverse Fourier transform. The field in the extended domain #2′ is then given by:14
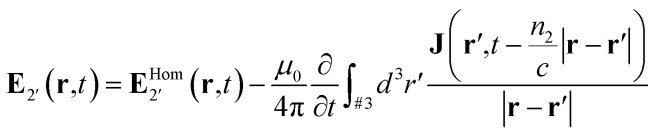


One can now apply the multipole decomposition developed in the ESI.† In particular the starting point is nearly identical to eqn (S2).† Repeating the same steps, and using the same approximations, one achieves the equivalent of eqn (S28):†15
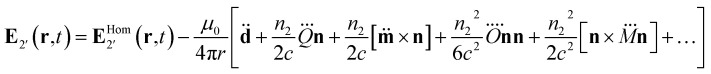
where *Q* is the electric quadrupole, *O* is the electric octupole moments and index 2′ designates the union of domains #2 and #3.

The only notable difference between this and the previous sections is the absence of the surface integrals (**U***etc.*) since the ‘current density’ here was explicitly constructed so that it vanishes outside domain #3. The application of the domain merge method of multipole analysis is given in the next subsection for the case of light propagating through the planar waveguide with a cylindrical air-filled inclusion.

#### 2.2.1 Domain merging approach applied to the scatterer or an inclusion in a planar waveguide

**Fig. 3:**
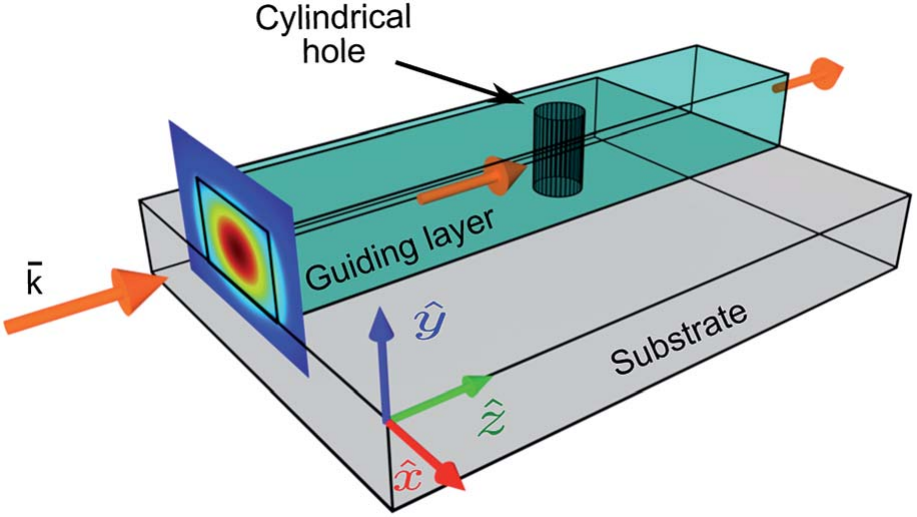
Illustration of the waveguide with cylindrical transverse cavity through the guiding layer. The width of the waveguide is 1 μm and the height is *t*_wg_ = 0.8 μm. The cross-section of the guided mode is shown in the input facet of the waveguide.

As an example, we consider the perturbed waveguide depicted in Fig. 3. Light is guided through a ridge dielectric waveguide made of a silicon-nitride (Si_3_N_4_) core (width of 1 μm, height of *t*_wg_ = 0.8 μm) placed on a silica substrate. We consider a telecommunication wavelength of *λ*_0_ = 1.55 μm, and refractive indices for silica and silicon-nitride as *n*_sub_ = 1.444 and *n*_wg_ = 1.997, respectively. The fundamental mode cross-section of the un-perturbed waveguide is shown in Fig. 3 for which the effective refractive index is *n*_eff_ = 1.74. Next, we study the perturbed waveguide. The perturbed waveguide is created by inserting the air-filled cylinder cavity through the waveguide core. It is important to study the inclusions of cylindrical shape since those shapes can be easily fabricated using focused ion beam (FIB) milling methods. By shaping the inclusion to different geometries, the multipoles will be affected as reported in ref. 18. The diameter of the cylinder is 100 nm, oriented perpendicular to the silica substrate. This cavity is illuminated by the mode of the un-perturbed waveguide.

To describe the scattering due to the perturbation into the free-space, the domain in the simulation needs to be truncated to reduce the computational complexity. This is performed by introducing layers of gradually increasing loss around the waveguide or by using PML boundary conditions. While this strategy allows us to simulate the propagation of light in the waveguide of finite size without un-physical reflections from the boundary, it also prevents one from knowing the far-field radiation due to cylindrical perturbation. Here, we show that the domain merging approach can be applied to determine the multipole representation of the perturbation.

We convert the field inside the cylinder into current density, **J**, as described by eqn (10). The multipole equivalents of the excitation due to hole perturbation can then be computed using eqn (S31)† for the electric dipole (see the ESI†):16
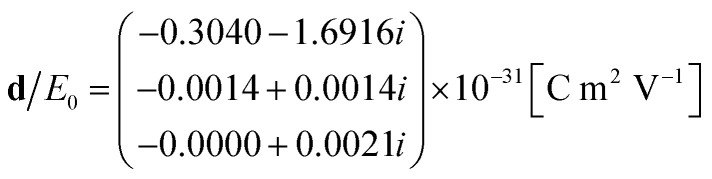
where *E*_0_ is the amplitude of the light coupled into the waveguide, using eqn (S19)† for the magnetic dipole (see the ESI†):17
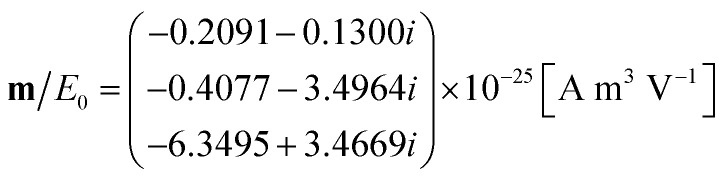
and using eqn (S32)† for the electric quadrupole (see the ESI†):18
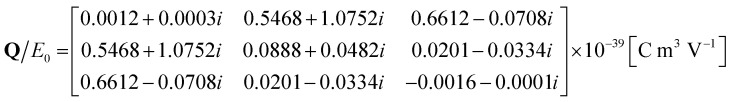


Using eqn (S68) from the ESI,† we calculate the power emitted by the electric dipole, magnetic dipole and electric quadruople for comparison. Emitted powers due to the electric dipole (*I*_d_), magnetic dipole (*I*_m_), and electric quadrupole (*I*_Q_) are normalized to the incident power *I*_in_ = 3*e*^13^ [W] and given by:19
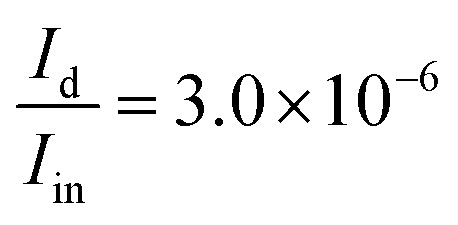
20
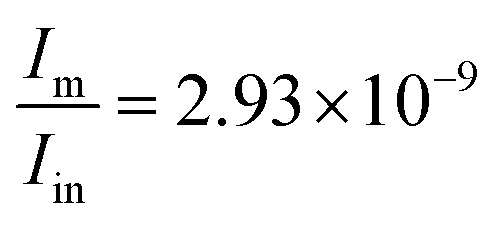
21
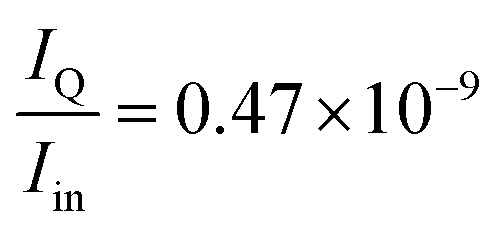


We note that the radiation from a small cavity in the waveguide can be described mostly as an electric dipole (embedded in the waveguide), with a small contribution from the magnetic dipole and electric quadrupole.

The described technique is useful when analyzing the illumination properties of different specific structures. As an example, we consider the emission (leakage) from a waveguide through a narrow cylindrical hole in it.

#### 2.2.2 Numerical proof-of-concept results

To prove our approach numerically, we built a numerical model utilizing the finite element method (FEM) of COMSOL Multiphysics 5.4. The guided mode was numerically calculated for a silicon nitride waveguide of rectangular cross-section, on a silica substrate, surrounded by air (using wave optics module). To induce scattering, an inclusion of cylindrical shape was removed from the waveguide core at the center of the waveguide. This resulted in scattering of light from the inclusion. The waveguide with the inclusion was embedded into a larger box with no excitation at any facets (and gradually increasing loss in the vicinity of all facets). The excitation in the cylinder, that matched either electric dipole or higher multipole terms, as well as their combinations was successively prescribed inside the cylinder embedded into the waveguide which led to scattering. The electric dipole and higher multipole equivalents of the cylinder have been calculated as outlined in the paper – in essence, this involved integrating the electric field inside the cylinder. We consider the single mode silicon nitride waveguide shown in Fig. 3 with a cylindrical inclusion with a diameter of *D* = *λ*/10 and illuminated by transverse electric (TE) polarized light at a wavelength *λ* = 1550 nm.

**Fig. 4:**
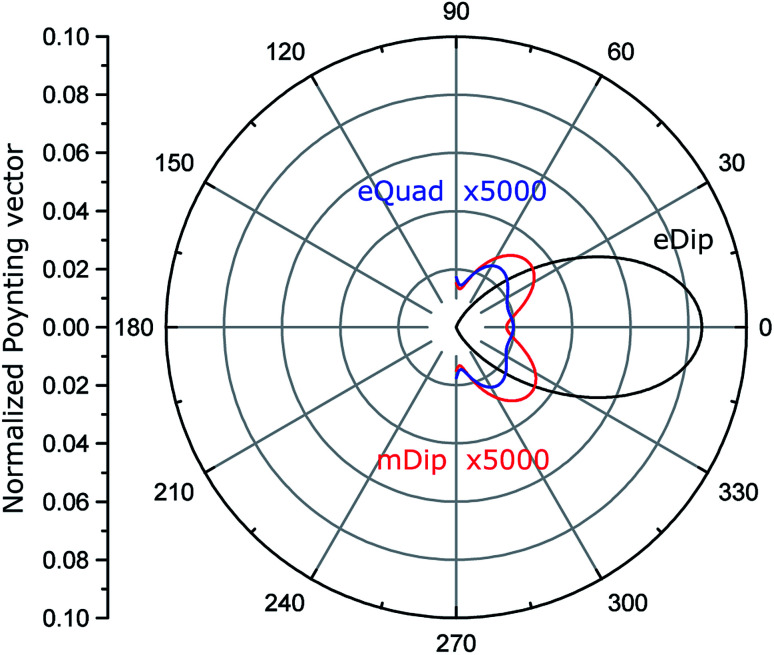
Normalized angular diagram in the *xy* plane. The electric quadrupole (eQuad) and magnetic dipole (mDip) are multiplied by 5000 for comparison.

**Fig. 5:**
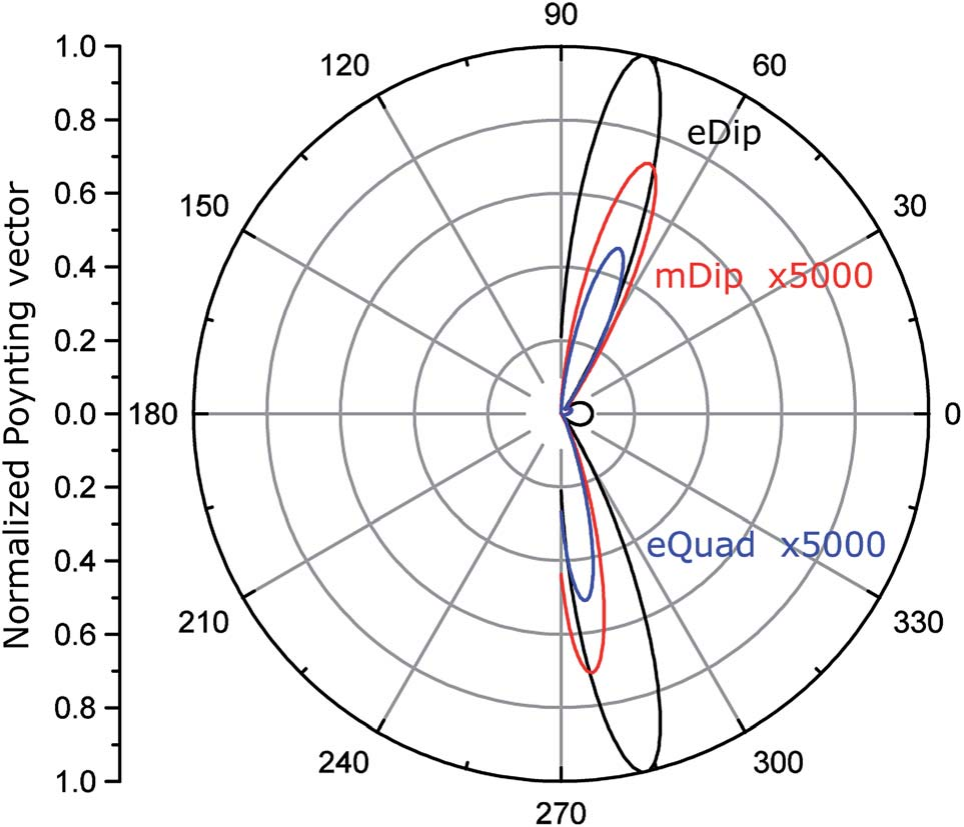
Normalized angular diagram in the *yz* plane. The electric quadrupole (eQuad) and magnetic dipole (mDip) are multiplied by 5000 for comparison.

Fig. 4 shows the calculated normalized to the maximum, angular diagrams of electric dipole (eDip), electric quadrupole (eQuad) and magnetic dipole (mDip) moments generated from the inclusion in the *xy* plane. Fig. 5 shows the analogous characteristics in the *yz* plane.

By analyzing the results presented in Fig. 4 and 5, we notice that the electric dipole moment is absolutely dominant in the field emitted from the hole.

## 3 Hollow transverse cavity in the waveguide as a single photon source

An in-depth understanding of the extended multipole decomposition of non-isolated sources of electromagnetic radiation is not only relevant for a microscopic description of fundamental phenomena, such as light propagation in a medium, but also for emerging applications such as atom-chip systems,^46^ for instance, the atomic clocks on a chip^47^ or sources of single photon emitters on a chip in all-dielectric systems. As compared to current on-chip single photon sources,^48^ controlling the shape and material of the inclusion made in the waveguide core, is a completely new elegant approach. To implement single photon emitters on a chip, we consider the ridge dielectric waveguide analyzed in Section 2 perturbed with the cylindrical inclusion (Fig. 3).

The cavity shown in Fig. 3 can be converted into a cylindrical distributed source, using the domain merging approach as described in Section 2. Generally speaking, our multipole decomposition for non-isolated sources of electromagnetic radiation is very useful for calculating the fields emitted outside the cavity beyond the waveguide boundaries. However, the parameters of the cavity can be tuned in such a way that the cavity-waveguide system will operate in a single photon emission regime. Having said that, here we do not deal with a quantum source involving an N-level system but rather, we consider a free photon gas. Our evaluation provides the estimation of leaked (emitted) energy and correspondingly the mean probability of it. Such an approach allows for the mathematical description of single photon emitters and, therefore, enables the design of on-chip sources in the mean probability sense. For this, we analyze the field inside the cavity in cylindrical coordinates (*r*, *φ*, *y*), where *y* is the axis of the cavity. Considering a cavity with *D* ≪ *λ*, the field inside the cavity is uniform in the cross-section (*r*, *φ*), and depends only on *y*. The diameter of the cavity is small compared to both (1) the wavelength in the waveguide *D* ≪ *λ*_wg_, and (2) the waveguide width. Therefore, we assume that the guided mode *E*_mode_ at the center of the waveguide is independent of *z* and *x* coordinates in a narrow area around the cavity. Thus, considering a transverse electric (TE) mode in the waveguide, the field inside the considered small cavity can be described as **E**_ins_ = **x̂***E*_*x*_(*z*). However, the emission process, in this case, has to be treated as spontaneous emission,^49^*i.e.*, as leakage of the photon gas from a volume. Analyzing such a system requires its quantization. The example of using quantization is given in ref. 50 containing N-level sources (such as a quantum dot) which is unnecessary here. Rather, we consider a box with a free photon gas, illuminated by the waveguide modes. Thus, we do not involve the quantum description, but only estimate the mean value of the photon number in a unit time interval. When discussing quantum sources, the statistical characteristics are in scope. In the literature, we find many papers underlying the non-Markovian statistics of photons illuminated by quantum dots.^51^ However, considering our free photon gas system, we assume that the photons obey just Markovian statistics since any emission does not depend on previous emission events.^52^ To demonstrate the anti-bunching feature,^53^ we calculate the angular diagrams shown in Fig. 4 and 5 which predict that the probability of emitting two photons during the same time intervals is negligible. A similar direction would be reduced by a factor of 0.5. A higher reduction of the bunching probability can be obtained with beam splitters.

The field inside such a narrow cavity can be taken approximately as *E*_ins_ ∼ *E*_mode_, see *e.g.*^54,55^ Thus the energy inside (*W*) can be estimated as *W* = *E*_mode_^2^*t*_wg_*D*^2^ and without actual quantization, we can evaluate this energy using the photon number *N*_ph_.22
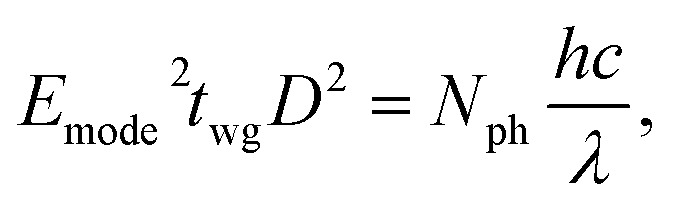
where *h* is Planck's constant.

It is important to estimate the probability of the photon emission through the open aperture of the cylindrical cavity during a period of 1 second. This probability is proportional to the ratio23
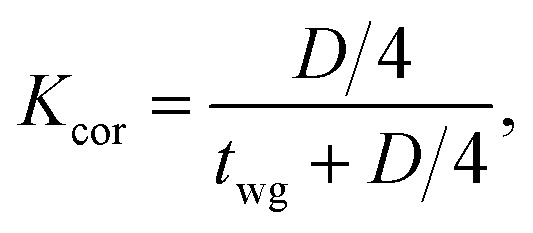
which is the ratio of the square of the open aperture (π*D*^2^/4) to the sum of the square of the side surface (π*t*_wg_*D*) and the square of the bottom aperture (π*D*^2^/4). The ratio in eqn (23) is enhanced by the reflection from the side and bottom surfaces of the cylindrical cavity. Therefore, the relation obtained can be corrected approximately as24
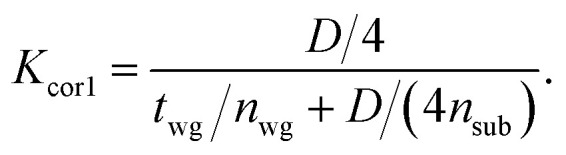


In addition, the *k*_*x*_ and *k*_*z*_ components of vector **k** have to be considered. In the case where *k*_*z*_/*k*_*x*_ is small, it will reduce the evaluated probability which can be estimated by the number of emitted photons *N*^e^_ph,s_. Inside the cavity, however, we have to take Heisenberg's principle into account which predicts the uncertainty of the momentum and the energy of the emitted photon *N*^e^_ph,s_ as well as diffraction and the broadening of the spectrum. In the present evaluation, we omit *k*_*z*_/*k*_*x*_ and Heisenberg's principle remain with25
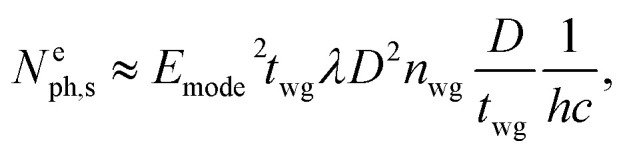
or26
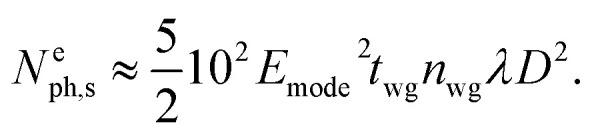


Thus, providing 
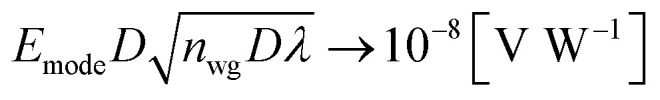
, we expect that this structure would emit 1 photon per 1 nanosecond, *i.e.*, acting as a single-photon emitter. Since the location of the emitter is distributed in the cylindrical cavity along *y*, our multipole decomposition of non-isolated sources of electromagnetic radiation is extremely useful for the analysis of the probability of photon emission in a specific direction for a broad range of applications.

**Fig. 6:**
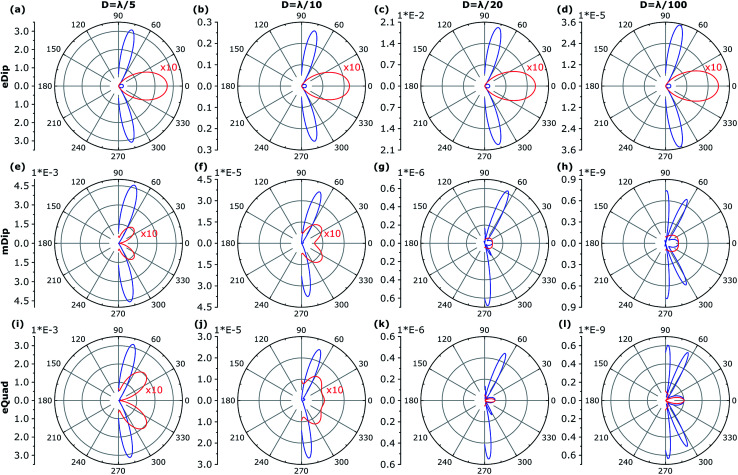
Angular diagrams for *D* = *λ*/(5, 10, 20, 100) (different diameters from each column as labeled above the columns) for fundamental TE. Some of the fields at the *xy* plane in the polar graph are multiplied by 10 for comparison. The blue curve shows the angular diagram in the *yz* plane and the red curve shows the angular diagram in the *xy* plane, respectively. The 1^st^ row shows the electric dipole (eDip), the 2^nd^ row shows the magnetic dipole (mDip), and the 3^rd^ row shows the electric quadrupole (eQuad) – as labeled in the *y*-axis of (a–i).

**Fig. 7:**
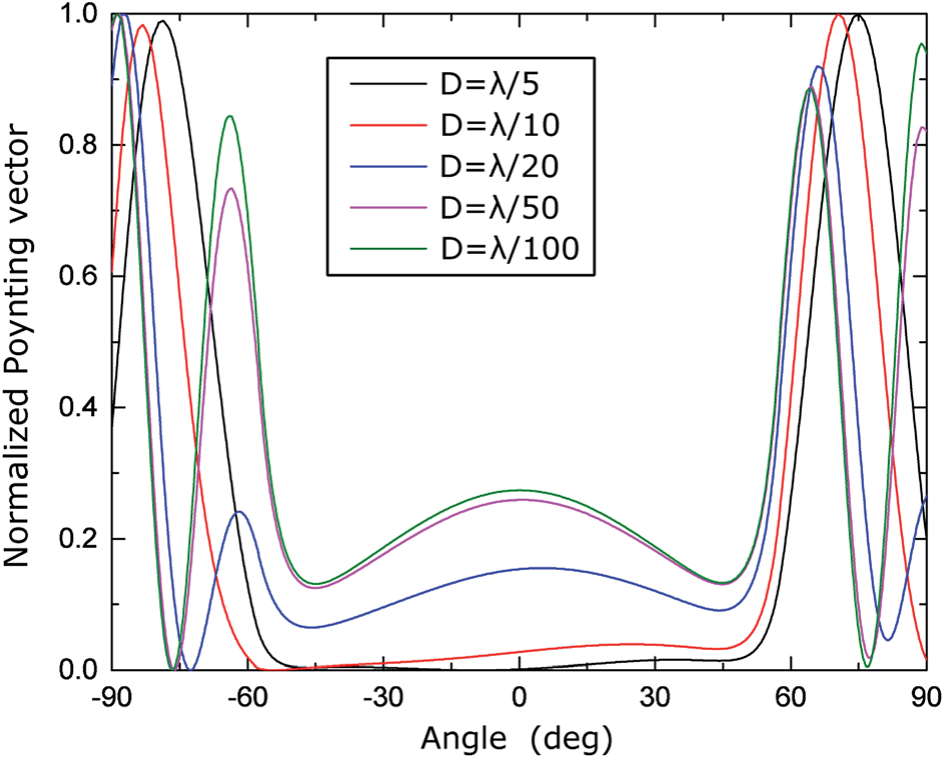
Calculated magnetic dipole for *D* = *λ*/(5, 10, 20, 50, 100) presented in a Cartesian coordinate plot.

The energy and the angular properties of the field emitted from the inclusion depends on the size of the inclusion. To explore this, we compare the angular diagrams of the emitted field while varying the diameter of the inclusion *D* from *λ*/5, *λ*/10, and *λ*/20 to *λ*/100, illuminated using *λ* = 1.55 μm. Fig. 6 shows the angular diagrams of multipole emitted power from the inclusion (cylindrical cavities) with diameters of *D* = *λ*/(5, 10, 20, 100). The blue curve shows the angular diagram in the *yz* plane, and the red curve shows the angular diagram in the *xy* plane, correspondingly. The 1^st^ row corresponds to the electric dipole (eDip), the 2^nd^ row corresponds to the magnetic dipole (mDip), and the 3^rd^ row corresponds to the electric quadrupole (eQuad) – as labeled in the left corner of each row. As expected, with narrowing the hole, the electric dipole predominates. It is also interesting to indicate the angular dependence of the irradiation by the electric dipole. Interestingly, we notice the appearance of additional diffraction maxima in case the diameter *D* becomes small. The asymmetry of diffraction maxima (for instance for *D* = *λ*/10) is determined by **k** = **k**_w_ ± **k**_il,m_ where **k**_w_ is the influence of the waveguide and **k**_il,m_ is the influence of the scattered fields from the cylinder. For a relatively large radius, the incident field on the cylinder is generated from the propagating modes in the waveguides as well as from the reflections of the scattered fields. As the radius becomes smaller, the scattered field magnitudes decrease, while the propagating modes do not experience a change. Therefore, the scattered field pattern evolves more like the fundamental electric and magnetic multipoles. This evolution, for instance, for the magnetic dipole calculated for different hole diameters is presented in Cartesian coordinates in Fig. 7.

## 4 Conclusion

To conclude, we present a formalism which allows analyzing the disturbance, such as a particle or inclusion, placed on, or enclosed by, a large system for single-photon source applications on a chip. In this case, currents and charges are not localized within the boundaries of particles leading to inaccuracy in the utilization of classical multipole decomposition. To address this challenge, we amended each multipole term. We note that these amendments become zero for closed systems. Based on Babinet's principle, we proposed an intuitively simple domain merging approach. To prove the proposed concept, we considered a CMOS compatible single mode silicon nitride ridge waveguide on a silica substrate with a perturbation of cylindrical shape and illuminated it with a TE fundamental mode. The multipole analysis showed that the equivalent source to the whole waveguide system can be represented by the electric dipole with directivity along the *x*-axis, while the contribution of electric quadrupoles and magnetic dipoles is minor. Changing the geometry of the inclusion will affect the contribution of multipoles to the directional scattering effect as was shown in our previous work.^18^ Our formalism enables studies of the light scattering effect by small perturbations, such as quantum dots, chemical analytes, nanolasers and others, in large non-isolated systems, such as waveguides and cavities.

## Conflicts of interest

There are no conflicts to declare.

## Supplementary Material

NA-003-D0NA00580K-s001
