# Supplementary material for: Non-isolated sources of electromagnetic radiation by multipole decomposition for photonic quantum technologies on a chip with nanoscale apertures
Source: Nanoscale Adv. 2020 Oct 8;3(1):190–7. doi: 10.1039/d0na00580k (PMC9417329; doi:10.1039/d0na00580k)
Supplement: NA-003-D0NA00580K-s001 [file NA-003-D0NA00580K-s001.pdf]

Electronic Supplementary Material (ESI) for Nanoscale Advances.
This journal is © The Royal Society of Chemistry 2020

Journal Name

ARTICLE TYPE

Cite this: DOI: 00.0000/xxxxxxxxxx

Non-isolated sources of electromagnetic radiation on a chip by multipole decomposition with nanoscale apertures - supplementary information

Yuriy A. Artemyev[a,b], Vassili Savinov[c], Aviad Katiyi[a], Alexander S. Shalin[b] and Alina Karabchevsky*[a]

S1 Detailed derivation of multipoles and amendments

Any electric and magnetic field can be represented by six quantities, however, only four of them are independent. Therefore, we can describe electric and magnetic fields using four quantities: the scalar potential (Φ), and the three components of the vector potential, ($\mathbf{A}$). Figure 1 (in main text) shows a particle of an arbitrary shape at the origin of coordinate system O. Assuming Lorenz gauge condition, retarded potentials of electromagnetic field produced by such arbitrary shaped source in the medium with permittivity of $\varepsilon\varepsilon_0$ (where ε_0 is electric constant and ε is dimensionless relative permittivity) and permeability $\mu\mu_0$. The $\mathbf{A}$ vector potential and Φ scalar potential are:

$$\Phi(\mathbf{R},t) = \frac{1}{4\pi\varepsilon\varepsilon_0}\int_V \frac{\rho\left(\mathbf{r}, t-\frac{|\mathbf{R}-\mathbf{r}|}{v}\right)}{|\mathbf{R}-\mathbf{r}|}dV \tag{S1}$$

$$\mathbf{A}(\mathbf{R},t) = \frac{\mu\mu_0}{4\pi}\int_V \frac{\mathbf{J}\left(\mathbf{r}, t-\frac{|\mathbf{R}-\mathbf{r}|}{v}\right)}{|\mathbf{R}-\mathbf{r}|}dV \tag{S2}$$

where $v = \frac{1}{\sqrt{\varepsilon\varepsilon_0\mu\mu_0}}$ is the speed of light in a medium, ρ is the electrical charge density, $\mathbf{r}$ is the distance vector to the volume dV of the particle and $\mathbf{R}$ is the distance vector to the observation point. We will denote modulus of vectors by usual letters: $r \equiv |\mathbf{r}|$, $R \equiv |\mathbf{R}|$.

Considering the field in the region $R \gg r$, we can expand $|\mathbf{R}-\mathbf{r}|$ into Taylor series. We use Einstein notation and take the sum over all pairs of repeated indices. Next, we consider the time dependence of potentials:

$$\begin{aligned} t - \frac{|\mathbf{R}-\mathbf{r}|}{v} &= t - \frac{R}{v}\sqrt{1-2\eta(\hat{\mathbf{r}}\cdot\hat{\mathbf{R}})+\eta^2} \\ \eta &\equiv r/R \end{aligned} \tag{S3}$$

For small η, we obtain:

$$\begin{aligned} J_i\left(\mathbf{r}, t-\frac{|\mathbf{R}-\mathbf{r}|}{v}\right) &= J_i(\mathbf{r},t') + \dot{J}_i(\mathbf{r},t')\frac{(\hat{\mathbf{r}}\cdot\hat{\mathbf{R}})}{v}\eta \\ &- \dot{J}_i(\mathbf{r},t')\frac{1}{2Rv}R^2\eta^2 + \dot{J}_i(\mathbf{r},t')\frac{(\hat{\mathbf{r}}\cdot\hat{\mathbf{R}})^2}{2Rv}R^2\eta^2 \\ &+ \ddot{J}_i(\mathbf{r},t')\frac{(\hat{\mathbf{r}}\cdot\hat{\mathbf{R}})^2}{2v^2}R^2\eta^2 + \dots \end{aligned} \tag{S4}$$

Substituting the definition of η into the series:

$$\begin{aligned} J_i\left(\mathbf{r}, t-\frac{|\mathbf{R}-\mathbf{r}|}{v}\right) &= J_i(\mathbf{r},t') + \dot{J}_i(\mathbf{r},t')\frac{r_j}{vR}R_j \\ &- \dot{J}_i(\mathbf{r},t')\frac{r^2}{2Rv} + \dot{J}_i(\mathbf{r},t')\frac{r_j r_k}{2R^3 v}R_j R_k \\ &+ \ddot{J}_i(\mathbf{r},t')t'\frac{r_j r_k}{2R^2v^2}R_jR_k + \dots \end{aligned} \tag{S5}$$

The series is considerably simplified by limiting the consideration to far-field (i.e. $\lambda v/cR \ll 1$ for all important wavelength components of the emitted radiation):

$$\begin{aligned} J_i(\mathbf{r},t'+\delta t) &= J_i(\mathbf{r},t') + \frac{\partial J_i(\mathbf{r},t')}{\partial t'}\delta t \\ &+ \frac{1}{2}\frac{\partial^2 J_i(\mathbf{r},t')}{\partial t'^2}\delta t^2 + \frac{1}{6}\frac{\partial^3 J_i(\mathbf{r},t')}{\partial t'^3}\delta t^3 + \dots \end{aligned} \tag{S6}$$

where $\delta t = t - R/v$ which is equivalent to:

$$\begin{aligned} J_i(\mathbf{r},t'+\delta t) &= J_i + \dot{J}_i\frac{R_j}{vR}r_j + \ddot{J}_i\frac{R_jR_k}{2v^2R^2}r_jr_k \\ &+ \dddot{J}_i\frac{R_jR_kR_m}{6v^3R^3}r_jr_kr_m + \dots \end{aligned} \tag{S7}$$

Where the overdot is the partial derivative over the retarded time.

Consider the Taylor series expansion of the function $1/|\mathbf{R}-\mathbf{r}|$:

$$\begin{aligned} \frac{1}{|\mathbf{R}-\mathbf{r}|} &= \frac{1}{R} + \frac{R_ir_i}{R^3} + \frac{3}{2R^5}R_iR_j \\ &- \frac{1}{3}\delta_{ij}R^2r_ir_j + \dots \approx \frac{1}{R} \end{aligned} \tag{S8}$$

[a] School of Electrical and Computer Engineering, Ben-Gurion University, Beer-Sheva, Israel.
[b] Department of Nano-Photonics and Metamaterials, ITMO University, St. Petersburg, Russia.
[c] Optoelectronics Research Centre and Centre for Photonic Metamaterials, University of Southampton, Southampton, UK.
* alinak@bgu.ac.il

We neglect high order terms except the zeroth-order term since all other terms can be suppressed by moving the detector shown in Fig. 1 (in main text) far enough from the source. This logic applies here and is technically correct. However, we note that in case of Equation (S7), one should include higher order terms since J_i could be an oscillatory function of time. In this case even a small change in the argument could lead to the large change in the function value.

Finally, for the vector potential, we obtain:

$$\mathbf{A}(\mathbf{R},t) = \frac{\mu\mu_0}{4\pi R}\left[\int_V \mathbf{J}\,dV + \frac{R_i}{vR}\int_V \dot{\mathbf{J}} r_i\,dV + \frac{R_j R_k}{2v^2R^2}\int_V \ddot{\mathbf{J}} r_j r_k\,dV + \frac{R_j R_k R_m}{6v^3R^3}\int_V \dddot{\mathbf{J}} r_j r_k r_m\,dV + \dots\right] \tag{S9}$$

A similar equation can be obtained for the scalar potential.

S1.1 Electric dipole moment and first amendment

Consider the integral $\int J_i\,dV$ in the first term in Equation (S9). To treat this term, we consider the continuity equation

$$\frac{\partial\rho}{\partial t} + \mathrm{div}\mathbf{J} = 0 \tag{S10}$$

utilizing the auxiliary equation we obtain

$$\nabla(\mathbf{J}r_i) = (\mathbf{J}\nabla)r_i + r_i(\nabla\mathbf{J}) = J_i - \dot{\rho} r_i. \tag{S11}$$

By integrating by parts the left side of Equation (S11) and rearranging terms, we obtain

$$\begin{aligned}\int_V J_i\,dV &= \int_V \dot{\rho} r_i\,dV + \int_V \nabla(\mathbf{J}r_i)\,dV \\ &= \dot{d}_i + \oint_S (\mathbf{n}_S\cdot\mathbf{J}) r_i\,dS = \dot{d}_i + U_i.\end{aligned} \tag{S12}$$

where d_i is i^{th} component of the electric dipole moment:

$$\mathbf{d} = \int_V \rho(\mathbf{r})\mathbf{r}\,dV \tag{S13}$$

The second term denoted by U_i, which is i^{th} component of some amendment vector, is obtained as a surface integral:

$$U_i = \oint_S (\mathbf{n}_S\cdot\mathbf{J}) r_i\,dS \tag{S14}$$

where $\mathbf{n}_S$ is the external normal vector to the surface S of the integration volume V. Namely this integral (and the following surface integrals) does provide the necessary amendment: For a closed system it turns to be zero, but for non-isolated system it gives a nonzero contribution.

S1.2 Electric quadrupole moment, magnetic dipole moment and second amendment

Consideration of the second term in the vector potential in Equation (S9) leads to:

$$R_j\int_V J_i r_j\,dV \tag{S15}$$

To treat it, we will use the following auxiliary expression:

$$\begin{aligned}R_j\int_V \nabla(\mathbf{J}r_i r_j)\,dV &= R_j\int_V (\dot{\rho} r_i r_j + J_i R_j r_j + r_i R_j J_j)\,dV \\ &= -\dot{Q}_{ij}R_j + 2\int_V J_i(R_j r_j)\,dV + \int_V \underbrace{[r_i(R_j J_j) - J_i(R_j r_j)]}_{\mathbf{b}(\mathbf{ac})-\mathbf{c}(\mathbf{ab})=[\mathbf{a}\times[\mathbf{b}\times\mathbf{c}]]}\,dV \\ &= -\dot{Q}_{ij}R_j + 2\int_V J_i(R_j r_j)\,dV + \mathbf{R}\times\int_V [\mathbf{r}\times\mathbf{J}]\,dV\end{aligned} \tag{S16}$$

From here we can obtain:

$$R_j\int_V J_i r_j\,dV = \frac{1}{2}\dot{Q}_{ij}R_j + [\mathbf{m}\times\mathbf{R}] + \frac{1}{2}U'_{ij}R_j \tag{S17}$$

where tensor $\hat{Q}$ is the electric quadrupole moment:

$$Q_{ij} = \int_v \rho(\mathbf{r}) r_i r_j\,dV \tag{S18}$$

vector $\mathbf{m}$ is the magnetic dipole moment:

$$\mathbf{m} = \frac{1}{2}\int_V [\mathbf{r}\times\mathbf{J}]\,dV \tag{S19}$$

The magnetic moment appears without involving magnetic permeability μ, but rather based only on the dielectric permittivity ε. For this reason, we obtain the resonance effect for high index dielectrics. We denote the second order amendment tensor $\hat{U}'$ as:

$$U'_{ij} = \oint_S (\mathbf{n}_S\cdot\mathbf{J}) r_i r_j\,dS \tag{S20}$$

S1.3 Electric octupole, magnetic quadrupole moments and third amendment

The third term in Equation (S9) leads to:

$$R_j R_k\int_V J_i r_j r_k\,dV \tag{S21}$$

By analogy with the previous cases, consider an auxiliary equation of the form:

$$\begin{aligned}R_j R_k\int_V \nabla(\mathbf{J}r_i r_j r_k)\,dV &= -R_j R_k\int_V \dot{\rho} r_i r_j r_k\,dV \\ &+ \int_V \left\{J_i(R_j r_j)(R_k r_k) + r_i(R_j J_j)(R_k r_k)\right\}dV \\ &+ \int_V r_i(R_j r_j)(R_k J_k)\,dV \\ &= -\dot{O}_{ijk}R_j R_k + 3R_j R_k\int_V J_i r_j r_k\,dV \\ &+ \int_V \left\{r_i(R_j J_j)(R_k r_k) - J_i(R_j r_j)(R_k r_k)\right\}dV \\ &+ \int_V \left\{r_i(R_j r_j)(R_k J_k) - J_i(R_j r_j)(R_k r_k)\right\}dV\end{aligned} \tag{S22}$$

Considering the third term in the last equation, the fourth term is treated similarly:

$$\int_V \left\{ r_i(R_jJ_j)(R_kr_k) - J_i(R_jr_j)(R_kr_k) \right\} dV$$
$$= \int_V \Big[\mathbf{R}\times[\mathbf{r}\times\mathbf{J}]\Big](\mathbf{rR})\,dV = R_jR_k\varepsilon_{ijq}\int_V \mathbf{r}\times\mathbf{J}_q r_k\,dV \qquad \text{(S23)}$$
$$= \mathbf{R}\times\left(\int_V \Big\{[\mathbf{r}\times\mathbf{J}]\otimes\mathbf{r}\Big\}\,dV\right)\mathbf{R}$$

where $\otimes$ is the tensor product. Summarizing Equations (S22) and (S23), we obtain the following result for Equation (S21):

$$R_jR_k\int_V J_ir_jr_k\,dV$$
$$= \frac{1}{3}\dot{O}_{ijk}R_jR_k - \mathbf{R}\times\left(\frac{2}{3}\int_V \Big\{[\mathbf{r}\times\mathbf{J}]\otimes\mathbf{r}\Big\}\,dV\right)\mathbf{R}$$
$$+\frac{1}{3}R_jR_k\oint_S(\mathbf{n}_S\cdot\mathbf{J})r_ir_jr_k\,dS \qquad \text{(S24)}$$
$$= \frac{1}{3}\dot{O}_{ijk}R_jR_k + [\mathbf{R}\times M\mathbf{R}] + \frac{1}{3}U''_{ijk}R_jR_k$$

Where, $\hat{O}$ is the electric octupole tensor:

$$O_{ijk} = \int_V \rho(\mathbf{r})r_ir_jr_k\,dV \qquad \text{(S25)}$$

where $\hat{M}$ is the magnetic quadrupole tensor:

$$M_{qm} = \frac{2}{3}\int_V [\mathbf{r}\times\mathbf{J}]_q r_m\,dV \qquad \text{(S26)}$$

and we denote $\hat{U}''$ as the amendment which is the third order tensor:

$$U''_{ijk} = \oint_S(\mathbf{n}_S\cdot\mathbf{J})r_ir_jr_k\,dS \qquad \text{(S27)}$$

Summarizing all above, we can write the multipole expansion of the vector potential:

$$\mathbf{A}(\mathbf{R},t) = \frac{\mu_0\mu}{4\pi R}\Bigg[\dot{\mathbf{d}} + \mathbf{U} + \frac{1}{2v}\ddot{Q}\mathbf{n} + \frac{1}{v}[\dot{\mathbf{m}}\times\mathbf{n}]$$
$$+\frac{1}{2v}\dot{U}'\mathbf{n} + \frac{1}{6v^2}\dddot{O}\mathbf{nn} + \frac{1}{2v^2}[\mathbf{n}\times\ddot{M}\mathbf{n}] \qquad \text{(S28)}$$
$$+\frac{1}{6v^2}\ddot{U}''\mathbf{nn} + \ldots\Bigg]$$

S1.4 Electric multipole moments

We briefly overview here the family of electric multipole moments:

$$q = \int_V \rho(\mathbf{r})\,dV \text{ — full charge}$$
$$d_i = \int_V \rho(\mathbf{r})r_i\,dV \text{ — electric dipole moment}$$
$$Q_{ij} = \int_V \rho(\mathbf{r})\,r_ir_j\,dV \text{ — electric quadrupole moment}$$
$$O_{ijk} = \int_V \rho(\mathbf{r})\,r_ir_jr_k\,dV \text{ — electric octupole moment}$$

In case of monochromatic time dependence

$$\rho(\mathbf{r},t) = \rho(\mathbf{r})e^{-i\omega t},$$

it can be useful to express electric multipole moments as functions of currents. From the continuity equation, we obtain:

$$\frac{\partial\rho}{\partial y} = -\mathrm{div}\mathbf{J} \quad\Rightarrow\quad \rho = \frac{1}{i\omega}\mathrm{div}\mathbf{J}. \qquad \text{(S29)}$$

using this relation, we can describe the electric multipole moments as function of the currents. The full charge is defined as:

$$q = \int_V \rho\,dV = \frac{1}{i\omega}\int_V \mathrm{div}\mathbf{J}\,dV = \frac{1}{i\omega}\oint_S(\mathbf{n}_S\mathbf{J})\,dS \qquad \text{(S30)}$$

Electric dipole moment is defined as:

$$d_i = \int_V \rho r_i\,dV = \frac{1}{i\omega}\int_V \mathrm{div}\mathbf{J}r_i\,dV$$
$$= \frac{1}{i\omega}\int_V \nabla(\mathbf{J}r_i)\,dV - \frac{1}{i\omega}\int_V(\mathbf{J}\nabla)r_i\,dV \qquad \text{(S31)}$$
$$= \frac{1}{i\omega}\oint_S(\mathbf{n}_S\mathbf{J})r_i\,dV - \frac{1}{i\omega}\int_V J_i\,dV$$

Electric quadrupole moment is defined as:

$$Q_{ij} = \int_V \rho r_ir_j\,dV = \frac{1}{i\omega}\int_V \mathrm{div}\mathbf{J}r_ir_j\,dV$$
$$= \frac{1}{i\omega}\int_V \nabla(\mathbf{J}r_ir_j)\,dV - \frac{1}{i\omega}\int_V(\mathbf{J}\nabla)r_ir_j\,dV \qquad \text{(S32)}$$
$$= \frac{1}{i\omega}\oint_S(\mathbf{n}_S\mathbf{J})r_ir_j\,dV - \frac{1}{i\omega}\int_V(J_ir_j + J_jr_i)\,dV.$$

Operating with quadrupole moments, it is usually preferred to deal with traceless tensors. The tensor, defined in Equation (S32), has a nonzero trace (denoted as qt). However, this is not important for our numerical treatment. If necessary, Equation (S32) can easily be converted into the traceless one using the well-known relation: $Q' = Q - qt * I$, where I is the diagonal unit tensor.

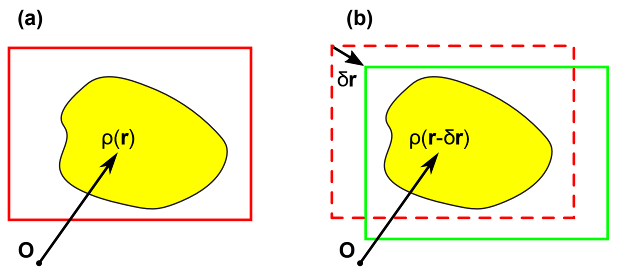

Fig. S1 (a) Homogeneous medium with charge density depending on point $\rho(\mathbf{r})$ and some arbitrary volume inside it. (b) The shift of the whole medium by some infinitesimal vector $\delta\mathbf{r}$ leads to the charge density at point $\mathbf{r}$ to become $\rho(\mathbf{r}-\delta\mathbf{r})$.

Further, for the electric octupole moment we develop:

$$\begin{aligned} O_{ijk} &= \int_V \rho r_i r_j r_k \, dV = \frac{1}{i\omega}\int_V \mathrm{div}\mathbf{J} r_i r_j r_k \, dV \\ &= \frac{1}{i\omega}\int_V \nabla(\mathbf{J} r_i r_j r_k)\, dV - \frac{1}{i\omega}\int_V (\mathbf{J}\nabla) r_i r_j r_k \, dV \\ &= \frac{1}{i\omega}\oint_S (\mathbf{n}_S \mathbf{J}) r_i r_j r_k \, dV \\ &- \frac{1}{i\omega}\int_V (J_i r_j r_k + r_i J_j r_k + r_i r_j J_k)\, dV \end{aligned} \tag{S33}$$

S1.5 Representation of multipole moments through polarization

For nanophotonics applications, it can be suitable to represent the multipole moments through polarization induced in dielectric[1,2]. For this, we first consider a homogeneous medium with a charge density continuously varying from point to point, and thus being a function of the radius vector. We choose some arbitrary volume V inside it (Fig. S1a), and shift the whole medium by some infinitesimal vector $\delta\mathbf{r}$ (Fig. S1b), to evaluate the change in charge density inside the volume.

The charge inside the volume V is

$$q = \int_V \rho(\mathbf{r}) dV, \tag{S34}$$

and the charge inside the volume after the shift of the medium is

$$\begin{aligned} q + \delta q &= \int_V [\rho(\mathbf{r}) + \delta\rho(\mathbf{r})]\, dV \\ &= \int_V \rho(\mathbf{r} - \delta\mathbf{r})\, dV = \int_V \left[\rho(\mathbf{r}) - \nabla\rho(\mathbf{r})\delta\mathbf{r}\right] dV \end{aligned} \tag{S35}$$

If the integrals over the arbitrary volumes are equal, then the integrand functions are also equal.

$$\delta\rho(\mathbf{r}) = -\nabla\rho(\mathbf{r})\delta\mathbf{r} \tag{S36}$$

Now, we can introduce the infinitesimally small polarization vector $\delta\mathbf{P}$. Since $\delta\mathbf{r}$ in Equation (S36) does not depend on $\mathbf{r}$, we manipulate with Equation (S36) as

$$\left[\nabla\rho(\mathbf{r})\right]\delta\mathbf{r} = \nabla\cdot\left[\rho(\mathbf{r})\,\delta\mathbf{r}\right] = \nabla\delta\mathbf{P} \tag{S37}$$

where we define:

$$\nabla\delta\mathbf{P} = -\delta\rho(\mathbf{r}) \tag{S38}$$

Since dielectrics are electroneutral, the initial charge inside the volume is zero. Therefore, the whole charge inside any volume is the induced charge. So we can write electric multipole moments through polarization using Equation (S38).

The electric dipole moment is defined as:

$$\begin{aligned} d_i &= \int_V \rho r_i dV = -\int_V r_i \mathrm{div}\mathbf{P}\, dV = \\ &- \int_V (\nabla\cdot\mathbf{P} r_i)\, dV + \int_V (\mathbf{P}\cdot\nabla r_i)\, dV = \\ &- \oint_S (\mathbf{n}_S\cdot\mathbf{P} r_i)\, dS + \int_V P_i\, dV \end{aligned} \tag{S39}$$

The electric quadrupole moment is defined as:

$$\begin{aligned} Q_{ij} &= \int_V \rho r_i r_j\, dV = -\int_V \mathrm{div}\mathbf{P} r_i r_j\, dV = \\ &- \int_V (\nabla\cdot\mathbf{P} r_i r_j)\, dV + \int_V (\mathbf{P}\nabla) r_i r_j\, dV = \\ &- \oint_S (\mathbf{n}_S\mathbf{P}) r_i r_j\, dS + \int_V (P_i r_j + P_j r_i)\, dV \end{aligned} \tag{S40}$$

The electric octupole moment is defined as:

$$\begin{aligned} O_{ijk} &= \int_V \rho r_i r_j r_k\, dV = -\int_V \mathrm{div}\mathbf{P} r_i r_j r_k\, dV = \\ &- \int_V (\nabla\cdot\mathbf{P} r_i r_j r_k)\, dV + \int_V (\mathbf{P}\nabla) r_i r_j r_k\, dV = \\ &- \oint_S (\mathbf{n}_S\mathbf{P}) r_i r_j r_k\, dS + \int_V (P_i r_j r_k + P_j r_i r_k + P_k r_i r_j)\, dV \end{aligned} \tag{S41}$$

We rewrite the magnetic multipole moments as functions of polarization. Using the continuity equation and Equation (S38):

$$\frac{\partial\rho}{\partial t} - \nabla\mathbf{J} = 0$$

$$\frac{\partial(\nabla\mathbf{P})}{\partial t} = \nabla\mathbf{J}$$

We replace the partial derivatives $\partial/\partial t$ with ∇, so

$$\mathbf{J} = \frac{\partial\mathbf{P}}{\partial t}$$

and assuming that the polarization $\mathbf{P}$ is time-harmonic, we obtain

$$\mathbf{J} = -i\omega\mathbf{P} \tag{S42}$$

Finally for the magnetic dipole we obtain:

$$\mathbf{m} = \frac{1}{2}\int_V [\mathbf{r}\times\mathbf{J}]\, dV = \frac{i\omega}{2}\int_V [\mathbf{P}\times\mathbf{r}]\, dV \tag{S43}$$

The magnetic quadrupole is defined as:

$$M_{ij} = \frac{2}{3}\int_V [\mathbf{r}\times\mathbf{J}]_i r_j\, dV = \frac{2i\omega}{3}\int_V [\mathbf{P}\times\mathbf{r}]_i r_j\, dV \tag{S44}$$

S1.5.1 Electric and magnetic fields

To obtain equations for fields, we consider the equation for vector potential:

$$\mathbf{A}(\mathbf{R},t) = \frac{\mu\mu_0}{4\pi R}\Big(\dot{\mathbf{d}} + \mathbf{U} + \frac{1}{2v}\ddot{\hat{Q}}\mathbf{n} + \\ + \frac{1}{v}\Big[\dot{\mathbf{m}}\times\mathbf{n}\Big] + \frac{1}{2v}\dot{\hat{U}}'\mathbf{n} + \frac{1}{6v^2}\dddot{\hat{O}}\mathbf{nn} \\ + \frac{1}{2v^2}\Big[\mathbf{n}\times\ddot{\hat{M}}\mathbf{n}\Big] + \frac{1}{6v^2}\ddot{\hat{U}}''\mathbf{nn} + \dots\Big) \tag{S45}$$

Magnetic field is expressed through the vector potential:

$$\mathbf{B} = \nabla\times\mathbf{A} \tag{S46}$$

and when we take rotor of the vector potential, we neglect the terms that occur due to factor $1/R$ in each term of the sum because of higher order of smallness (where ε_{ijk} denotes the Levi-Civita symbol),

$$\nabla\times\frac{1}{R}\mathbf{f}(t') = \mathbf{e}_i\varepsilon_{ijk}\Big(-\frac{R_j}{R^3}f_k(t') - \frac{R_j}{cR^2}\dot{f}_k(t')\Big) \\ = \frac{1}{R^2}[\mathbf{f}\times\mathbf{n}] + \frac{1}{vR}[\dot{\mathbf{f}}\times\mathbf{n}] \approx \frac{1}{vR}[\dot{\mathbf{f}}\times\mathbf{n}] \tag{S47}$$

We write down the expression for the rotor of each component of the sum in Equation (S45):

$$[\nabla\times\dot{\mathbf{d}}] = \mathbf{e}_i\varepsilon_{ijk}\nabla_j\dot{d}_k = \mathbf{e}_i\varepsilon_{ijk}\frac{\partial\dot{d}_k}{\partial t'}\nabla_j\Big(t-\frac{R}{c}\Big) \\ = -\frac{1}{v}\mathbf{e}_i\varepsilon_{ijk}n_j\ddot{d}_k = -\frac{1}{v}[\mathbf{n}\times\ddot{\mathbf{d}}] \tag{S48}$$

$$[\nabla\times\mathbf{U}] = -\frac{1}{v}[\mathbf{n}\times\dot{\mathbf{U}}] \tag{S49}$$

$$\frac{1}{2v}[\nabla\times\ddot{\hat{Q}}\mathbf{n}] = \frac{1}{2v}\mathbf{e}_i\varepsilon_{ijk}\Big(-4-\frac{1}{v}\dddot{\hat{Q}}_{ks}n_jn_s \\ + \ddot{Q}_{ks}\frac{\delta_{js}}{R} + \ddot{Q}_{ks}\frac{R_jR_s}{R^3}\Big) \approx -\frac{1}{2v^2}\Big[\mathbf{n}\times\dddot{\hat{Q}}\mathbf{n}\Big] \tag{S50}$$

$$\frac{1}{v}[\nabla\times[\dot{\mathbf{m}}\times\mathbf{n}]] = -\frac{1}{v^2}[\mathbf{n}\times[\ddot{\mathbf{m}}\times\mathbf{n}]] \tag{S51}$$

$$\frac{1}{2v}\Big[\nabla\times\Big(\dot{\hat{U}}'\mathbf{n}\Big)\Big] \approx -\frac{1}{2v^2}\Big[\mathbf{n}\times\ddot{\hat{U}}'\mathbf{n}\Big] \tag{S52}$$

$$\frac{1}{6v^2}\Big[\nabla\times\Big(\dddot{\hat{O}}\mathbf{nn}\Big)\Big] \approx -\frac{1}{6v^3}\mathbf{e}_i\varepsilon_{ijk}\ddddot{O}_{kst}n_jn_sn_t \\ = -\frac{1}{6v^3}\Big[\mathbf{n}\times\ddddot{\hat{O}}\mathbf{nn}\Big] \tag{S53}$$

$$\frac{1}{2v^2}\Big[\nabla\times\Big[\mathbf{n}\times\ddot{\hat{M}}\mathbf{n}\Big]\Big] = \frac{1}{2v^2}\mathbf{e}_i\varepsilon_{ijk}\nabla_j\,\varepsilon_{klm}\frac{R_l}{R}\ddot{M}_{ms}\frac{R_s}{R} \\ = \frac{1}{2v^2}\mathbf{e}_i\varepsilon_{ijk}\varepsilon_{klm}\Big(\frac{R_s}{R^2}\delta_{jl}\ddot{M}_{ms} + \frac{R_l}{R^2}\delta_{js}\ddot{M}_{ms} \\ -2\frac{R_jR_lR_s}{R^5}\ddot{M}_{ms} - \dddot{M}_{ms}\frac{R_jR_lR_s}{cR^3}\Big) \\ \approx -\frac{1}{2v^3}\Big[\mathbf{n}\times\Big[\mathbf{n}\times\dddot{\hat{M}}\mathbf{n}\Big]\Big] \tag{S54}$$

$$\frac{1}{6v^2}\Big[\nabla\times\Big(\ddot{\hat{U}}''\mathbf{nn}\Big)\Big] \approx -\frac{1}{6v^3}\Big[\mathbf{n}\times\dddot{\hat{U}}''\mathbf{nn}\Big] \tag{S55}$$

Thus, the magnetic field, while remaining only terms of the first order of smallness, is:

$$\mathbf{B} = \frac{\mu\mu_0}{4\pi R v}\Big([\ddot{\mathbf{d}}\times\mathbf{n}] + [\dot{\mathbf{U}}\times\mathbf{n}] + \frac{1}{2v}\Big[\dddot{\hat{Q}}\mathbf{n}\times\mathbf{n}\Big] \\ + \frac{1}{v}[\mathbf{n}\times[\mathbf{n}\times\ddot{\mathbf{m}}]] + \frac{1}{2v}\Big[\ddot{\hat{U}}'\mathbf{n}\times\mathbf{n}\Big] + \frac{1}{6v^2}\Big[(\ddddot{\hat{O}}\mathbf{n})\cdot\mathbf{n}\times\mathbf{n}\Big] \\ + \frac{1}{2v^2}\Big[\mathbf{n}\times\Big[\dddot{\hat{M}}\mathbf{n}\times\mathbf{n}\Big]\Big] + \frac{1}{6v^2}\Big[\dddot{\hat{U}}''\mathbf{nn}\times\mathbf{n}\Big]\Big) \tag{S56}$$

In this form, the only remaining term is the first order of smallness. It can be seen that this equation can be written in short form:

$$\mathbf{B} = \frac{1}{v}[\dot{\mathbf{A}}\times\mathbf{n}] \tag{S57}$$

which corresponds to a vector $\mathbf{H}$ in a plane wave[3].

The electric field is expressed in terms of the potentials as

$$\mathbf{E} = -\dot{\mathbf{A}} - \nabla\Phi \tag{S58}$$

Finally, remaining only terms of the first order of smallness, we obtain:

$$\mathbf{E} = \frac{1}{4\pi R v^2\varepsilon\varepsilon_0}\Big([[\ddot{\mathbf{d}}\times\mathbf{n}]\times\mathbf{n}] + [[\dot{\mathbf{U}}\times\mathbf{n}]\times\mathbf{n}] \\ + \frac{1}{2v}\Big[\Big[\dddot{\hat{Q}}\mathbf{n}\times\mathbf{n}\Big]\times\mathbf{n}\Big] + \frac{1}{v}[\ddot{\mathbf{m}}\times\mathbf{n}] \\ + \frac{1}{2v}\Big[\Big[\ddot{\hat{U}}'\mathbf{n}\times\mathbf{n}\Big]\times\mathbf{n}\Big] + \frac{1}{6v^2}\Big[\Big[\ddddot{\hat{O}}\mathbf{nn}\times\mathbf{n}\Big]\times\mathbf{n}\Big] \\ + \frac{1}{2v^2}\Big[\mathbf{n}\times\dddot{\hat{M}}\mathbf{n}\Big] + \frac{1}{6v^2}\Big[\Big[\dddot{\hat{U}}''\mathbf{nn}\times\mathbf{n}\Big]\times\mathbf{n}\Big]\Big) \tag{S59}$$

Then, Equation (S59) also can be written in a simple form:

$$\mathbf{E} = v[\mathbf{B}\times\mathbf{n}] = [[\dot{\mathbf{A}}\times\mathbf{n}]\times\mathbf{n}] \tag{S60}$$

which also corresponds to an electric field $\mathbf{E}$ in a plane wave[3].

Both equations for $\mathbf{B}$ (S56) and $\mathbf{E}$ (S59) are the same as plane wave illumination. After the terms $\sim 1/R$, we consider the field at distances much larger compared to the system and at a sufficient distance from the source arbitrary shaped wavefront, and this can be locally considered as a plane wave.

Assuming plane wave illumination, $E(\mathbf{r},t) = E_0e^{i(\omega t-\mathbf{k}\cdot\mathbf{r})}$ where

$\mathbf{k}$ is the wave vector of the incident wave,

$$\omega = k_0 c = kv \tag{S61}$$

$$c = 1/\sqrt{\mu_0 \varepsilon_0} \tag{S62}$$

$$v = 1/\sqrt{\mu \mu_0 \varepsilon \varepsilon_0} \tag{S63}$$

$$k = k_0 \sqrt{\mu \varepsilon} \tag{S64}$$

then we can write the equation for the scattered electric field in the following form:

$$\begin{aligned}\mathbf{E} = \frac{k^2}{4\pi\varepsilon_0\varepsilon}\frac{e^{ikR}}{R}\Bigg(& [\mathbf{n}\times[\mathbf{d}\times\mathbf{n}]] + \frac{i}{kv}[\mathbf{n}\times[\mathbf{U}\times\mathbf{n}]] \\ & + \frac{ik}{2}[\mathbf{n}\times[\hat{Q}\mathbf{n}\times\mathbf{n}]] + \frac{1}{v}[\mathbf{m}\times\mathbf{n}] \\ & + \frac{1}{2v}[\mathbf{n}\times[\hat{U}'\mathbf{n}\times\mathbf{n}]] + \frac{k^2}{6}[\mathbf{n}\times[\mathbf{n}\times\hat{O}\mathbf{nn}]] \\ & + \frac{ik}{2v}[\mathbf{n}\times\hat{M}\mathbf{n}] + \frac{ik}{6v}[\mathbf{n}\times[\mathbf{n}\times\hat{U}''\mathbf{nn}]\Bigg)\end{aligned} \tag{S65}$$

The factor e^{ikR} appears in this equation, as the phase shift of the scattered wave between the points $\mathbf{r}$ and $\mathbf{R}$.

S1.5.2 Intensity

In this section we provide a tool for analysing the 'strengths' of different multipole excitations which together represent the current density within an arbitrarily chosen volume (see Fig. 1 in main text). The problem we are solving is that one cannot directly compare different multipoles, e.g. the electric dipole and quadrupole moments, since they have different units. Nevertheless, all multipoles represent an excitation in the system, and thus there should be a way of comparing them. Here we propose to use the power of the light that would be emitted by the different multipoles, if there were no other currents outside the considered volume. Thus electric quadrupole excitation, for example, could be said to be 'stronger' than electric dipole excitation, if the power emitted by the quadrupole (in all directions), was greater than that of the electric dipole.

Using the Poynting vector definition[4], the energy radiated Π into solid angle $d\Omega$ can be expressed as:

$$d\Pi = \frac{1}{2}\sqrt{\frac{\varepsilon\varepsilon_0}{\mu\mu_0}}|\mathbf{E}|^2 R^2\, d\Omega \tag{S66}$$

The total energy scattered on such system per unit time (intensity of scattered light) can be obtained by integrating over all solid angles:

$$I = \int_\Omega d\Pi$$

To perform the integral above, we average $d\Pi$ over all angles. Therefore, the total energy can be obtained by multiplication of the average power, $\overline{d\Pi}$, by the solid angle of a sphere:

$$I = 4\pi\overline{d\Pi} \tag{S67}$$

In $d\Pi$ only $\mathbf{n}$, a unit vector into an observation point, depends on a direction. By averaging, we use several useful and well-known relations (see, e.g.,[5]).

Eventually, we obtain the expression for the intensity of light scattered per unit time:

$$\begin{aligned} I = & \frac{k^4}{12\pi v\mu\mu_0\varepsilon^2{\varepsilon_0}^2}|\mathbf{d}|^2 + \frac{k^2}{12\pi v\varepsilon^2{\varepsilon_0}^2}|\mathbf{U}|^2 \\ & + \frac{k^6}{32\pi v\mu\mu_0\varepsilon^2{\varepsilon_0}^2}\left(\frac{1}{5}Q_{ij}Q_{ij}^* - \frac{1}{15}Q_{ii}Q_{jj}^*\right) \\ & + \frac{k^4}{12\pi v\varepsilon\varepsilon_0}|\mathbf{m}|^2 + \frac{k^4}{32\pi v\varepsilon\varepsilon_0}\left(\frac{1}{5}U'_{ij}U'^*_{ij} - \frac{1}{15}U'_{ii}U'^*_{jj}\right) \\ & + \frac{k^8}{288\pi v\mu\mu_0\varepsilon^2{\varepsilon_0}^2}\left(\frac{8}{105}O_{ijk}O_{ijk}^* - \frac{2}{105}O_{ijj}O_{ikk}^*\right) \\ & + \frac{k^6}{32\pi v\varepsilon\varepsilon_0}\left(\frac{1}{5}M_{ij}M_{ij}^* - \frac{1}{15}M_{ii}M_{jj}^*\right) \\ & + \frac{k^6}{288\pi v\varepsilon\varepsilon_0}\left(\frac{8}{105}U''_{ijk}U''^*_{ijk} - \frac{2}{105}U''_{ijj}U''^*_{ikk}\right)\end{aligned} \tag{S68}$$

Basically, different terms depend differently on optical contrast of the medium ε.

Notes and references

1 A. B. Evlyukhin, C. Reinhardt, E. Evlyukhin and B. N. Chichkov, *JOSA B*, 2013, **30**, 2589–2598.

2 A. B. Evlyukhin, T. Fischer, C. Reinhardt and B. N. Chichkov, *Physical Review B*, 2016, **94**, 205434.

3 L. D. Landau and E. Lifshitz, *Course of theoretical physics*, 1975, 281.

4 C. F. Bohren and D. R. Huffman, *Absorption and scattering of light by small particles*, John Wiley & Sons, 2008.

5 J. A. Schouten, *Tensor analysis for physicists*, Courier Corporation, 1989.
